# Multiple Impact Damage in GLARE Laminates: Experiments and Simulations

**DOI:** 10.3390/ma14247800

**Published:** 2021-12-16

**Authors:** Sang-Eui Lee, Dong-Uk Kim, Yong-Jun Cho, Hyoung-Seock Seo

**Affiliations:** 1 Department of Mechanical Engineering, Inha University, Incheon 22212, Korea; selee@inha.ac.kr (S.-E.L.); 22201203@inha.edu (Y.-J.C.); 2 School of Naval Architecture and Ocean Engineering, University of Ulsan, Ulsan 44610, Korea; orrist@ulsan.ac.kr

**Keywords:** GLARE 5-2/1, aluminum 2024-T3, multiple impact, BVID, CVID, FEM (finite element method), failure criteria, VUMAT

## Abstract

Experiments and finite element simulations for multiple impact were performed on GLARE 5-2/1 and aluminum 2024-T3. Experiments were conducted on aluminum 2024-T3 and GLARE 5-2/1 at diverse impact energies to produce BVID (barely visible impact damage) and CVID (clearly visible impact damage). The finite element model was developed for multiple impact analysis using ABAQUS software and was confirmed by comparing the finite element analysis outcomes with experimental results. The two- and three-dimensional failure criteria model was applied to predict multiple impact behavior such as load-time history, maximum deflection-impact energy history, and damage progression. In addition, a user subroutine VUMAT was created to represent a three-dimensional progressive failure and was linked with ABAQUS. FEM results showed good agreement with experimental data.

## 1. Introduction

Fiber metal laminates (FMLs) are a hybrid composite material composed of metallic layers and fiber-reinforced composite laminates. Since this combination can achieve a good mechanical performance and damage durability such as tension, fatigue, and impact, it has been widely used in aircraft, aerospace, ships, cars, and so on. Aluminum and titanium are the most widely used metal for FML, and the fibers usually adopted are carbon, glass, and Kevlar fibers. Commonly, FMLs such as CARALL (carbon fiber aluminum laminates) and GLARE (S2-glass fiber-reinforced aluminum laminates) are composed of alternating layers of unidirectional or bi-directional fiber-reinforced prepregs with aluminum alloy sheets. Especially, in the case of GLARE, it was developed at Delft University in the Netherlands [1,2,3,4]. It has been used successfully for the Airbus A380 and has shown outstanding mechanical performances compared to fiber-reinforced composite laminates or aluminum alloys [5]. In the case of aircraft structure in transportation, the most important issues are safety related to impact resistance, damage durability, and endurance after single or multiple impacts. Continuous impact damages of aircraft structures can happen by collision between cargo vehicles, dropped tools during maintenance, hail and bird strikes, and lightning strikes [6]. In particular, multiple impact can cause more severe damages to a structure. When the first impact is applied on the structure, the load-carrying capacity is dropped severely. After applying the second impact sequentially, the residual strength is more decreased critically. Especially, in the case of a composite structure, it is hard to investigate impact damage. If the second impact is applied on the same location of structure continuously without recognizing the first impact damage, laminate stiffness, strength, and the load-bearing ability related to damage durability and tolerance will be decreased dramatically [7,8,9,10,11,12,13]. Despite these reasons, very few research works are found in literature that investigate the multiple impact behavior of GLARE laminates [14].

Moriniere et al. [14] investigated the damage of GLARE by repeated impact load. In this study, impact was applied repeatedly until penetrating GLARE specimens. They found that fracture at the composite layer occurred along with cracking of the top aluminum, and delamination was observed between the composite layer and aluminum layer. In this study, experimental results showed composite layers disturbed the perforation of aluminum, while the aluminum delayed delamination growth in the composite. Rajkumar et al. [15] analyzed the residual tensile strength of glass fiber metal composites after repeated low-impact through drop-weight impact tests. The results appeared to be the ultimate tensile strength, failure strain, and ductility of FMLs decreased in the beginning but no longer changed after applying repeated impact. Yao et al. [16] investigated the multiple impact behavior of FMLs consisting of carbon fiber-reinforced layers and aluminum sheets. Through experiment and numerical simulation on VUMAT, they studied the influence of multiple impact, load sequence, and metal layer distribution on multiple impact behavior and damage characteristics of FMLs. In this literature, Hashin failure criteria [17] and Yeh delamination damage criteria [16] were used to simulate failure modes and damage evolution of composite laminates. The results revealed that multiple impact under lower energy and small initial impact energy can cause minor damage in FMLs.

Seo et al. [18] and Yu et al. [19] developed a finite element model (FEM) for GLARE 4-3/2, GLARE 5-2/1, and CARALL under single impact using the explicit finite element code ABAQUS. Their study developed an explicit user-defined material subroutine (VUMAT) that implemented the Hashin 3D failure criteria to predict damage initiation and examined the damaged material’s constitutive response. Seo et al. investigated the differences between predicted and measured load-time history and maximum permanent impactor dent depth in diverse impact damage cases such as dent damage, crack damage, and perforation damage for diverse GLARE laminates. Yu et al. compared the differences between GLARE and CARALL under single impact through experiments and finite element analysis. In this literature, CARALL represented the better impact resistance than GLARE due to the high strength and stiffness of carbon fiber-reinforced plastic.

For composite materials, previous researchers focused on the investigation of interlaminar damage and development of effective numerical models for predicting multiple impact damages. Amouzou et al. [11] and Found et al. [13] performed multiple impact tests and investigated the effects of multiple impact on CFRP (carbon fiber-reinforced polymers). The results showed impact strength was improved at 45° and 60° of fiber orientations and the second impact showed a significant reduction of impact force and an increase in the impact duration. Mittelman [20] studied the effects of residual strength after impact at various absorbed energy composites. The results exhibited multiple was found to affect the interlaminar residual strength. In the case of internal damage related to interlaminar residual strength, damages as a result of multiple impact were found in the plies of matrix cracking and in between plies of delamination.

This paper mainly addresses investigating the low velocity impact behavior of GLARE laminate under multiple impact with diverse total energy. Experiments and finite element simulations were performed to investigate and predict over a range of impact damages such as BVID (8 J (2 × 4 J), 16 J (2 × 8 J)) and CVID (26 J (2 × 13 J)) of GLARE 5-2/1. GLARE 5-2/1 consisting of two aluminum layers and one S2-glass composite layer and aluminum 2024-T3 were tested under different impact cases in a drop weight test machine. To examine the performance of multiple impact of GLARE 5-2/1, aluminum 2024-T3 was compared as a reference material through the experiment. Experimental results were used to validate the simulation predictions related to peak impact force, deflection, and the internal damage area. For simulation work, ABAQUS/Explicit was applied to investigate the multiple impact behavior and failure mechanisms of GLARE and aluminum. To consider progressive failure and damage evolution of aluminum sheets and composite laminate, a three-dimensional, explicit, vectorized, user-defined material subroutine (VUMAT) was developed based on the mechanical constitutive behavior and Hashin’s failure criteria. Additionally, the three-dimensional failure model was compared with a two-dimensional failure model built in to ABAQUS by evaluating peak force, central deformation, and damage progression in the composite layers.

## 2. Experimental Work

### 2.1. Materials and Specimens

GLARE laminate can be manufactured to match a variety of applications by changing the fiber/resin system, the alloy type and thickness, stacking sequence, fiber orientation, and the surface pretreatment technique [5]. The mechanical property of GLARE laminates can be diversified according to the relative thickness of aluminum sheet and glass fiber/epoxy layers, the number of layers in the laminate, and the fiber volume fraction. GLARE laminates are manufactured by bonding together unclad metal sheets with composite prepreg using a hot press or an autoclave [3]. Before bonding lay-up, the metal layer surfaces are treated to improve the adhesion to prepreg. After the lay-up procedure, the GLARE laminate is cured in an autoclave. There are several kinds of GLARE laminates with different laminate lay-up and fiber orientation. Especially, GLARE 5 with fiber orientations of [0°/90°/90°/0°] has the excellent impact performance depending on a high strain-rate strengthening of the glass fibers.

**Figure 1:**
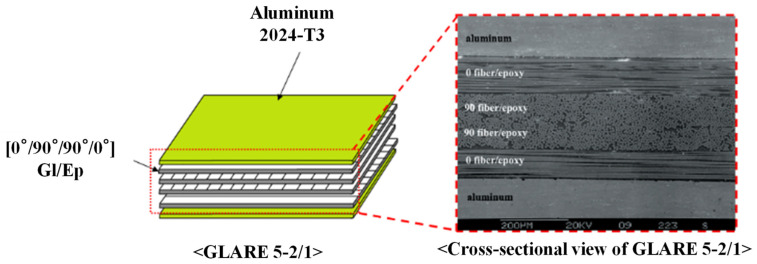
Configuration of GLARE 5-2/1 in detail.

Aluminum and GLARE laminate used in the present experiment were aluminum 2024-T3 and GLARE 5-2/1 provided by Aviation Equipment, Inc. (Costa Mesa, CA, USA). Figure 1 shows the composition of GLARE 5-2/1 tested in the experimental works. As shown in Figure 1, the cross section of GLARE 5-2/1 consists of two layers of aluminum 2024-T3 and one layer of S2 glass/epoxy composite with [0°/90°/90°/0°]. The average thicknesses of different layers were measured using optical micrographs. The average thicknesses of the aluminum layer and composite layer in GLARE 5-2/1 were 0.489 mm and 0.584 mm, respectively. Thus, the total laminate thickness of GLARE 5-2/1 was 1.562 mm. On the other hand, the average thicknesses of the aluminum 2024-T3 specimen was 1.60 mm.

### 2.2. Impact Test Procedure

**Figure 2:**
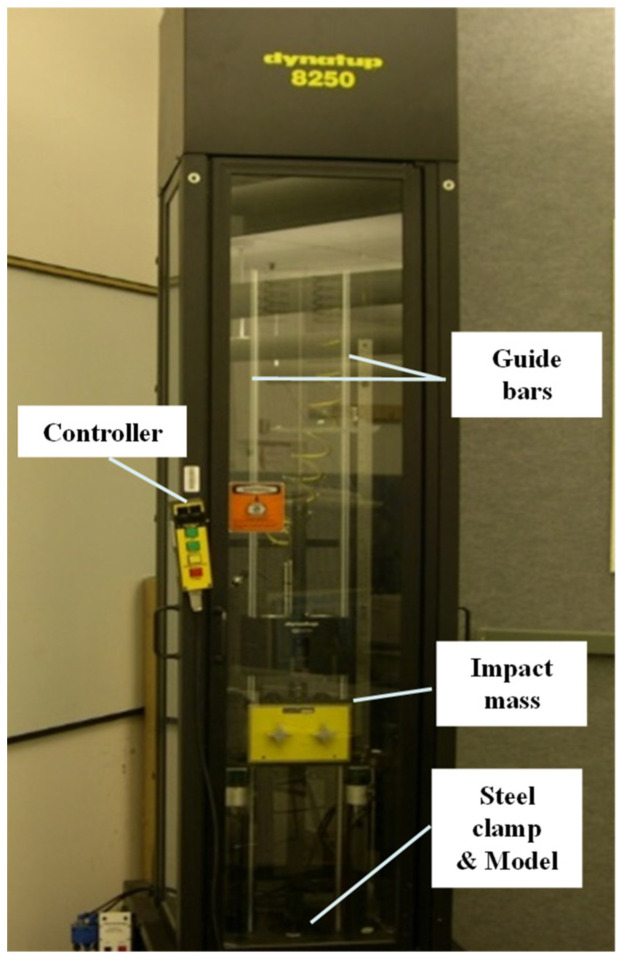
Dynatup model 8250 instrumented drop weight impact tower.

As shown in Figure 2, a Dynatup Model 8250 (Instron, Norwood, MA, USA) drop-weight impact tower was used for multiple impact test below 5 m/s of low impact velocity. Aluminum 2024-T3 was tested as a reference for comparison with GLARE 5-2/1. Data were collected during impact tests on a PC-based data acquisition system (Dynatup GRC 930-I) with a photodiode velocity detector. After just applying the impact, pneumatic rebound breaks were activated to push up and hold the impactor assembly in place so that the specimen was not subjected to multiple impacts. For the multiple impact test, all specimens were cut to sizes of 76.2 mm × 76.2 mm by water jet. After cutting the specimens, the pre-damage occurrence was carefully detected. The specimens were clamped between two steel plates with a 114.3 mm × 114.3 mm, square shape. The steel plates had a circular opening with a 31.7-mm diameter at the center. To create diverse impact damages such as BVID and CVID, impact energies of 8 J (2 × 4 J), 16 J (2 × 8 J), and 26 J (2 × 13 J) were performed by changing the drop height in low-velocity multiple impact tests. To achieve multiple impact damage, the steel impactor of the semi-spherical end with a diameter of 12.7 mm and mass of 5.6 kg was dropped two times on the same points of aluminum 2024-T3 and GLARE 5-2/1. After impact testing, specimens were carefully removed to investigate the damage.

## 3. FE simulation for Multiple Impact

### 3.1. Development of Finite Element Model

Numerical simulation of multiple impact was performed in ABAQUS/Explicit by applying different multiple impact energy levels. The simulated impact energies were the same as those used in the experiment. In the finite element model, two different multiple impact energies were considered, corresponding to BVID of 8 J (2 × 4 J) and 16 J (2 × 8 J). The same dimension of the specimen used in the experiment was adopted to develop the finite element model of GLARE 5-2/1. The general contact algorithm of ABAQUS/Explicit was applied for multiple impact simulation of GLARE 5-2/1. To generate the stable time increment during impact simulation, the time increment was decided by (density/elastic modulus)^0.5^. This condition was needed to have the stable integration process in dynamic analysis. The finite element model of GLARE 5-2/1 consisted of two aluminum 2024-T3 layers and one S2-glass/epoxy composite layer. The material behavior of aluminum layer was assumed to be elasto-plastic with an isotropic hardening. The glass/epoxy composite layer was modeled with two- and three-dimensional failure criteria of Hashin failure criteria [17]. Especially, for developing a three-dimensional failure model of the glass/epoxy composite layer, the user material subroutine (VUMAT) of ABAQUS was built to calculate and degrade the stiffness matrix [21]. To develop the damage model of the composite laminate for impact behavior, there were several criteria of the maximum stress criteria: Tsai-Wu [22], Hashin [17,23], Hou [24], and Puck [25] with VUMAT user subroutine. Saniuste et al. [26] and Li et al. [27] concluded the Hashin failure criteria exhibited appropriate results closest to the experiment data. Especially, the Hashin failure criteria showed the proper damage progression result to predict the damage area of composite laminates under low velocity impact.

A hexahedral solid element (C3D8R) was used in the simulation for the aluminum layer, and a hexahedral solid element (C3D8R) and hexahedral shell continuum element (SC8R) were used for the glass/epoxy composite layer, respectively. In a dynamic impact simulation, each element experiences excessive distortion. Thus, hourglass control was used to prevent this problem. When elements experience distortion by excessive impact, hourglassing can be a critical problem with reduced-integration elements such as C3D8R and SC8R in stress/displacement analyses [28]. Since the elements have only one integration point, it is possible for them to distort in such a way that the strains calculated at the integration point are all zero, which leads to uncontrolled distortion of the mesh [28]. Therefore, proper hourglass control should be used for impact simulation.

**Figure 3:**
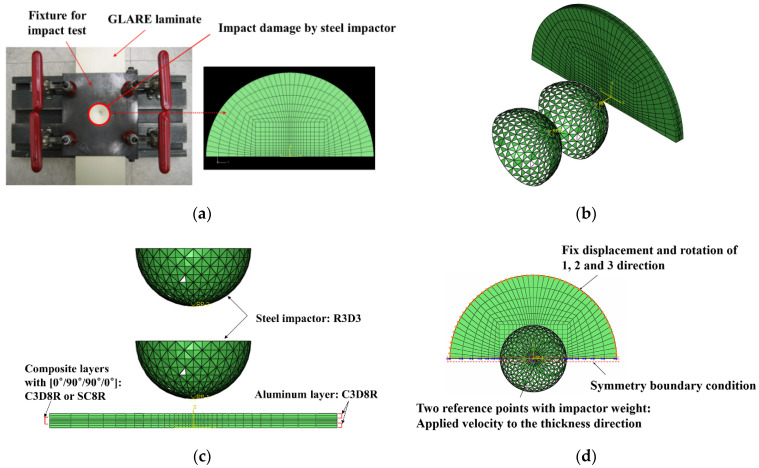
Finite element model and boundary conditions for GLARE 5-2/1: (**a**) finite element model and clamping fixture, (**b**) cross view of mesh geometry with multiple hemispherical impactors, (**c**) side view of mesh geometry with multiple hemispherical impactors, (**d**) boundary conditions of finite element model.

Figure 3a–d shows the mesh geometry and boundary condition used in the finite element modeling. As shown in Figure 3a, the mesh of the GLARE laminate was modeled based on the size of the real impact fixture hole. Figure 3b,c shows the cross view and the front view of multiple impact models. As seen in Figure 3b, two impactors were developed to apply two impacts. They contact each other when they are simulated to drop at different time intervals, but there is no interaction between these two impactors. They were only used to separately apply two impacts on the GLARE laminate. Figure 3d shows the boundary condition of the mesh geometry of GLARE 5-2/1. Only 1/2 of the GLARE laminate was modeled with the appropriate symmetric boundary conditions applied, as shown in Figure 3d. The displacements of all the edges of the mesh were fixed in the x, y, and z directions. The displacement and rotation of the impactor were only fixed in the x and y directions. Material, damage, and failure properties [4,5] of aluminum 2024-T3 and S2 glass fiber-reinforced epoxy layers used in the FEM simulation are summarized in Table 1 and Table 2, respectively.

### 3.2. Development of Damage Progression for Multiple Impact

ABAQUS offers continuum shell elements with a three-dimensional geometry of solid elements. This shell element gives an efficient method for computational simulations, but it is still locally planar [25]. Under the plane stress assumption, all through-thickness normal and shear components of the stress tensor (*σ*_13_, *σ*_23_, *σ*_33_) can be negligible. Therefore, such a system can make a continuum shell element available in the simulation for thin composite laminates. This assumption is reasonable for most thin composite structures, but it may not be an acceptable assumption in some specific cases such as thick composite structures or dynamic impact analysis. In our multiple impact analyses, it will produce non-trivial through-thickness stresses since the impact direction is parallel to the shell element normal vector. However, the solid element considering through-thickness stress is believed to complement the lack of a shell element.

Normally, continuum shell elements built in ABAQUS are widely used to analyze the composite materials to represent the progressive damage and failure of finite element model [28]. As discussed previously, since basic shell elements are not enough to simulate impact situation, a vectorized user material subroutine (VUMAT) was coded to represent damage progression and failure mode of three-dimensional solid elements of composite materials. This VUMAT based on displacement changes is working in ABAQUS/Explicit processor. In the VUMAT, Hashin failure criteria indicated in terms of the strain tensor (*ε_ij_*) and experimental failure strain (*ε_ij_^init^)* in Equations (1)–(4) appeared to represent four modes of composite damage with each mode represented by its own normalized failure variable (*f^i^*) [22]. These failure strains can be from general failure stress measurements through the inner product with respect to the corresponding components of the material compliance tensor. The Hashin criteria allow for four modes of composite damage with each mode represented by its own normalized failure variable (*f^i^*):

- Fiber Tension (FT) Failure Mode (*ε*_11_ > 0):
  (1) $$f^{ft}={\left(\frac{\varepsilon_{11}}{\varepsilon_{11+}^{init}}\right)}^{2}+\frac{1}{{\varepsilon_{12}^{init\;}}^{2}}\left(\varepsilon_{12}^{2}+\varepsilon_{13}^{2}\right)\geq 1$$
- Fiber Compression (FC) Failure Mode (*ε*_11_ < 0):
  (2) $$f^{fc}=\frac{-\varepsilon_{11}}{\varepsilon_{11-}^{init}}\geq 1$$
- Matrix Tension/Shear (MT) Failure Mode (*ε*_22_ *+ ε*_33_ > 0):
  (3) $$f^{mt}=\frac{1}{{\varepsilon_{22+}^{init\;}}^{2}}{\left(\varepsilon_{22}+\varepsilon_{33}\right)}^{2}+\frac{1}{{\varepsilon_{12}^{init\;}}^{2}}\left(\varepsilon_{23}^{2}+\varepsilon_{12}^{2}+\varepsilon_{13}^{2}-\varepsilon_{22}\varepsilon_{33}\right)\geq 1$$
- Matrix Compression (MC) Failure Mode (*ε*_22_ *+ ε*_33_ < 0):
  (4) $$f^{mc}=\frac{1}{\varepsilon_{22-}^{init}}\left[{\left(\frac{\varepsilon_{22-}^{init}}{2\varepsilon_{12}^{init\;}}\right)}^{2}-1\right]\left(\varepsilon_{22}+\varepsilon_{33}\right)+\frac{1}{{\varepsilon_{12}^{init\;}}^{2}}\left[\frac{\varepsilon_{22}^{2}+\varepsilon_{33}^{2}}{2}+\varepsilon_{23}^{2}+\varepsilon_{12}^{2}+\varepsilon_{13}^{2}\right]\geq 1$$

As seen in Equations (1)–(4), damage is assumed to occur in composite, when the summation of each directional strain at specific nodes of elements exceeds unity. This failure criterion includes all directional strain tensor for fiber and matrix. In the case of two-dimensional failure methodology in ABAQUS, since the out-of-plane shear strains (*ε*_13_ and *ε*_23_) are deleted, the out-of-plane normal strain (*ε*_33_) is obtained for explicit analysis:

(5) $$\varepsilon_{33}=\frac{1}{E_{22}}\left(\sigma_{33}-\nu_{23}\sigma_{22}\right)-\frac{\nu_{12}}{E_{11}}\sigma_{11}$$

**Figure 4:**
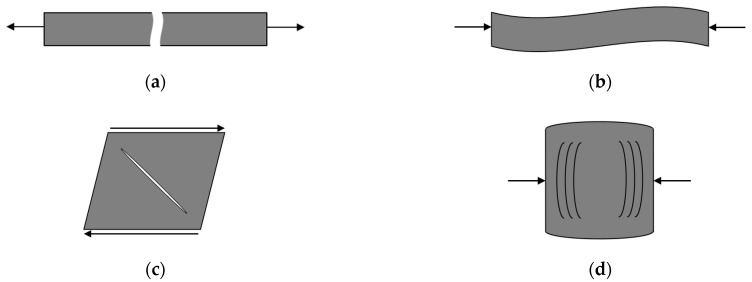
The Hashin composite-failure modes represented by their own normalized failure variable ($f^{i}$): (**a**) fiber tension mode, (**b**) fiber compression mode, (**c**) matrix tension/shear mode, (**d**) matrix compression mode.

The above form assumes the transverse isotropic property of the composite with a plane formed by the 2–3 axis showing the plane of isotropy. Since the composite has the anisotropy property, all calculations by the VUMAT worked on the local coordinate system. This is meaningful to provide a consistent analysis when the local principal material directions are not aligned with the global coordinate system. By the external load, when failure has just occurred in any given mode, the material property was degraded by the damage mechanics approached proposed by Matzenmiller et al. [29]. This damage system related to the four failure modes, which are illustrated schematically in Figure 4, decreases the load resistance of the area of the composite. Since the finite element model cannot reduce the physical area directly, an internal damage state variable (*w^i^*) operates to reduce the damage area by modifying the material’s elastic constants. As a result, the tensional stress components and a strain softening constitutive model are decreased by the damage in the material at a specific point. When the variables in the Hashin failure criteria, as seen in Equations (1)–(4), pass a value of one, the internal damage state of material accompanies a similar rate-based model suggested by Iannucci et al. [30]. The rate of damage propagation in the material at the current time step (${\dot{w}}^{i}\left(t+\Delta t\right)$ is decided by the damage state variable of the previous time step (*w^i^*(*t*)) and the current strain state (*ε*(*t + Δt*)) as well as the constant damage rate terms caused by crack occurrence and growth (*Ω_0_* and *Ω_1,_* respectively). The explicit equation for the damage propagation rate is shown in Equation (6) where the superscript (*i*) corresponds to each unique Hashin failure mode in Equations (1)–(4):

(6) $${\dot{w}}^{i}\left(t+\Delta t\right)=\Omega_{0}+\Omega_{1}w^{i}\left(t\right)\left[\left(\frac{\varepsilon_{kl}\left(t+\Delta t\right)\varepsilon_{kl}\left(t+\Delta t\right)}{\varepsilon_{mn}^{init}\varepsilon_{nm}^{init}{\left(1-w^{i}\left(t\right)\right)}^{2}}\right)-1\right]$$

In the Equation (6), the strain state at failure state at failure (*ε_ij_^init^*) is revised by the previous damage state (*w^i^*(*t*)). This covers the limited value of strain interrelated to composite damage decreases with the increase of damage density. Because the interval of the time step increment is so small, the degradation rate of Equation (6) is assumed to have the linear shape. Therefore, the damage state variable at the current time step (${\dot{w}}^{i}\left(t+\Delta t\right)$) can be influenced from the previous damage state and the damage growth rate:

(7) $$w^{i}\left(t+\Delta t\right)=w^{i}\left(t\right)+{\dot{w}}^{i}\left(t+\Delta t\right)\Delta t\;w^{i}\in \left[0,1\right)$$

In this case, the damage state variables are used to degrade the composite elastic constant, as seen in Table 3, and they should keep an upper limit that is less than one to avoid computational problems during inversion. Fiber failure modes (*f^ft^*, *f^fc^*) involved in the damage can affect the decrease of the modulus in the fiber direction (*E*_11_) along with the in-plane shear modulus and Poisson’s ratio (*G*_12_ and *v*_12,_ respectively). On the other side, the matrix failure mode (*f^mt^*, *f^mc^*) reduces all elastic constants except for the modulus in the local fiber direction. The composite material compliance (*S*(*t*)) at any time step can be obtained from Equations (8) and (9), where the linear elastic material response for an undamaged laminate is obtained by establishing all damage state variables to zero.

(8) $$S(t)=\left[\begin{array}{cccccc} \frac{1}{d_{1}E_{11}} & \frac{-1}{d_{3}}\left(\frac{v_{12}}{E_{11}}\right) & \frac{-1}{d_{3}}\left(\frac{v_{12}}{E_{11}}\right) & 0 & 0 & 0\\ \frac{-1}{d_{3}}\left(\frac{v_{12}}{E_{11}}\right) & \frac{1}{d_{2}E_{22}} & \frac{-1}{d_{2}}\left(\frac{v_{23}}{E_{22}}\right) & 0 & 0 & 0\\ \frac{-1}{d_{3}}\left(\frac{v_{12}}{E_{11}}\right) & \frac{-1}{d_{2}}\left(\frac{v_{23}}{E_{22}}\right) & \frac{1}{d_{2}E_{22}} & 0 & 0 & 0\\ 0 & 0 & 0 & \frac{1}{d_{3}G_{12}} & 0 & 0\\ 0 & 0 & 0 & 0 & \frac{1}{d_{3}G_{12}} & 0\\ 0 & 0 & 0 & 0 & 0 & \frac{2\left(1+d_{2}v_{23}\right)}{d_{2}E_{22}} \end{array}\right]$$

(9) d1=1−wft1−wfcd2=1−wmt1−wmcd3=1−wft1−wfc1−wmt1−wmc

In this constitutive model, the transversely isotropic feature of material is maintained following the onset of damage. At the current time step, the material compliance is just set up. Then, the current stress state in reduced index form can be decided from Equations (6)–(10) through inversion. In the case of non-zero damage state variables, the stresses transferred by a damaged element may be smaller than those of the corresponding undamaged element assumed by the damage mechanics.

(10) $$\begin{array}{ll} \vec{\sigma }\left(t+\Delta t\right) & =C\left(t+\Delta t\right)\vec{\varepsilon }\left(t+\Delta t\right)\\ C\left(t+\Delta t\right) & =S^{-1}\left(t+\Delta t\right) \end{array}$$

**Figure 5:**
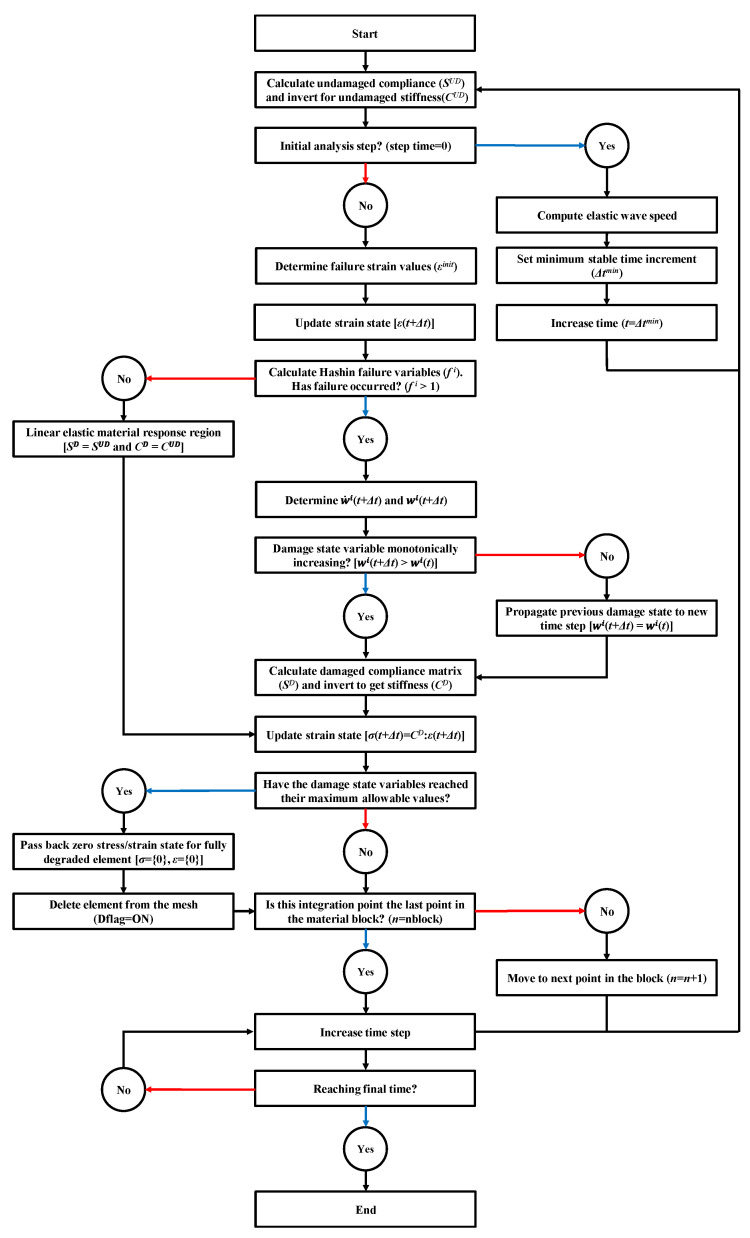
VUMAT flowchart for damage progression of composite in ABAQUS.

The whole procedure of damage mechanics in Equations (1)–(10) in the ABAQUS 6.11/Explicit processor is shown in Figure 5.

## 4. Result and Discussion

### 4.1. Experimental Results

**Figure 6:**
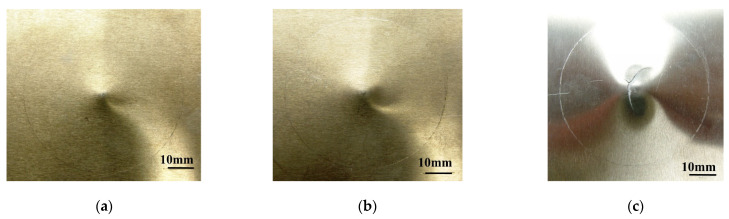
Multiple impact damage of aluminum 2024-T3 at (**a**) BVID (8 J (2 × 4 J)), (**b**) BVID (16 J (2 × 8 J)), and (**c**) CVID (26 J (2 × 13 J)).

**Figure 7:**
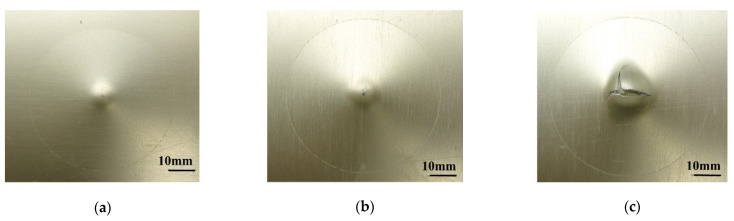
Multiple impact damage of GLARE 5-2/1 at (**a**) BVID (8 J (2 × 4 J)), (**b**) BVID (16 J (2 × 8 J)), (**c**) CVID (26 J (2 × 13 J)).

**Figure 8:**
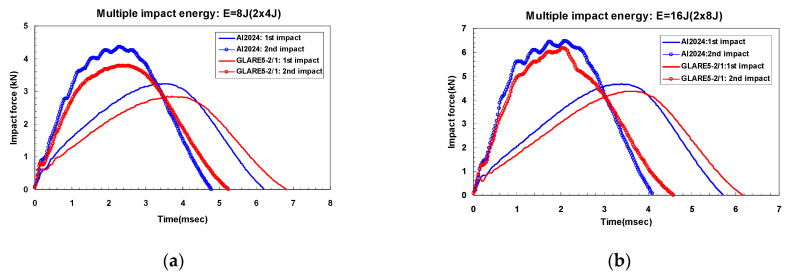
Impact force-time curves of aluminum 2024-T3 and GLARE 5-2/1 for multiple impact BVID cases: (**a**) 8 J (2 × 4 J); (**b**) 16 J (2 × 8 J).

Figure 6a,b and Figure 7a,b show the dent damage (BVID) caused by multiple impact in aluminum 2024-T3 and GLARE 5-2/1, respectively. The crack damage (CVID) caused by multiple impact is shown in Figure 6c and Figure 7c. In the case of CVID, the crack was created in the outer aluminum layer on the non-impacted side of GLARE 5-2/1 along the 0-degree and 90-degree fiber directions. Figure 8 shows experimental results for impact force-time history of aluminum 2024-T3 and GLARE 5-2/1. In the impact force-time curves of aluminum 2024-T3 at 8 J and 16 J impact levels (BVID), the maximum impact force was about 3.2 kN and 4.4 kN and the contact time was about 6.2 ms and 5.7 ms, respectively, during the first 4 J and first 8 J impact events. Then the impact force increased but contact duration decreases during 2nd-4 J and 2nd 8 J impact events. As shown in Figure 8a,b, after the first and the second impacts, the plastic deformation was observed through the prolonged stable impact force. The phenomena were investigated in the BVID case of aluminum 2024-T3. In the case of GLARE 5-2/1, the impact force-time curve after the second impact showed the different scenarios at 8 J and 16 J impact cases. In the impact force-time curves of GLARE 5-2/1 at 8 J and 16 J impact levels, the observed maximum impact force was about 2.6 kN and 4.2 kN and the contact time was about 6.8 ms and 6.2 ms, respectively, during the first 4 J and first 8 J. As shown in Figure 8a,b, after applying the first 4 J and first 8 J impacts, GLARE 5-2/1 exhibited a similar plastic deformation behavior as aluminum 2024-T3. This indicated that the aluminum sheet of GLARE 5-2/1 had the impact resistance for applied impact energy. After the second 4 J impact, aluminum and GLARE 5-2/1 showed the similar tendency as the first impact case. However, GLARE 5-2/1 displayed a little sharp load drop after the second 8 J impact, as shown in Figure 8b. This separate load drop was considered to point the delamination and failure caused by the progressive damage evolution of the composite layer during the second impact event [6]. Additionally, in the case of stiffness, both GLARE 5-2/1 and aluminum 2024-T3 showed an increase of stiffness after the second impact compared to that of the first impact from the slope of the impact force-time curve. This means the strain hardening caused by plastic deformation of aluminum layer can strengthen the impact resistance after the first impact [16]. The magnitude of slope was almost the same for aluminum and GLARE and it showed aluminum can work dominantly under impact force.

**Figure 9:**
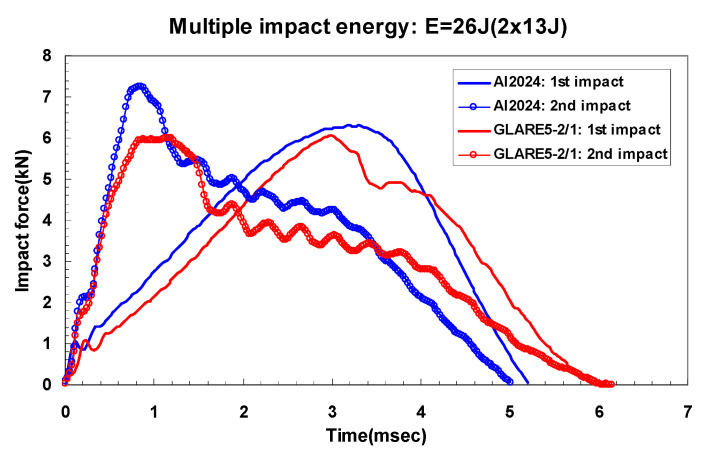
Impact force-time curves of aluminum 2024-T3 and GLARE 5-2/1 for multiple impact CVID case: 26 J (2 × 13 J).

Different damage morphology of aluminum 2024-T3 and GLARE 5-2/1 at 26 J (CVID) can be observed from Figure 9. As shown in Figure 9, the impact peak load in the second 13 J impact for aluminum 2024-T3 and GLARE 5-2/1 showed a similar tendency to increase compared with that in second 4 J and second 8 J impacts. After applying the first 13 J impact, aluminum 2024-T3 appeared to have plastic deformation behavior, but at the second 13 J impact, the impact force was decreased sharply, caused by crack on the non-impacted side of aluminum. After applying the first 13 J impact, GLARE 5-2/1 showed the combined damage behavior of plastic deformation and failure at composite layers, as shown in Figure 9. Until the impact force reached a peak point, the aluminum sheet of GLARE absorbed the impact energy at the peak contact time of 3 ms. After passing 3 ms, it had no longer enough strength for absorbing impact energy. Then, the impact energy was transferred to the S2 glass fiber layers, and this caused the internal damage such as delamination of composite layers. The phenomena could be used to explain the sudden drop in the load-time curve after the peak value at the first 13 J impact, as shown in Figure 9. Regarding the damage morphologies of GLARE 5-2/1 after the second 13 J impact, the evident crack aligned with the fiber directions of [0°/90°/90°/0°] and fiber fracture could be investigated on the composite layers of GLARE. After applying the second impact, the tendency of stiffness from load-time curve under CVID exhibited the similar result to that of the BVID case.

**Figure 10:**
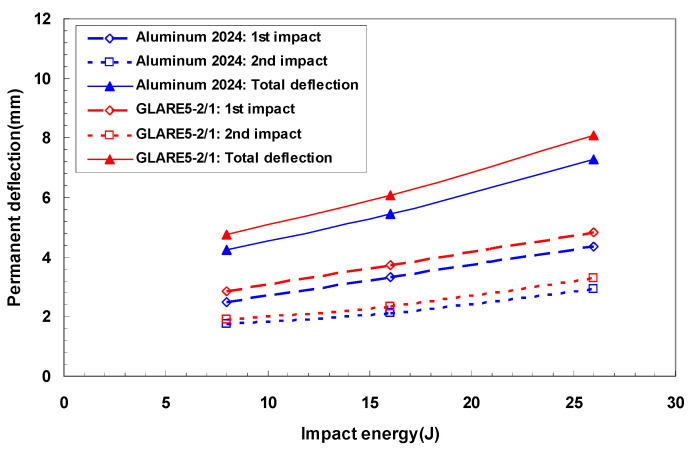
Irreversible central deflection as a function of the impact energy.

Figure 10 exhibits the irreversible central deflection of aluminum 2024-T3 and GLARE 5-2/1 as a function of impact energy after multiple impact. GLARE 5-2/1 showed the similar dent depth as aluminum 2024-T3 after the first impact. Since the aluminum sheet of GLARE 5-2/1 alleviated impact energy, there was a little difference of the permanent depth between aluminum and GLARE at the first impact case. However, after the second impact, GLARE 5-2/1 had a slightly deeper dent than aluminum 2024-T3. This was affected by the occurrence of the internal damage of the S2 glass fiber composite layers. Finally, the total dent depth between GLARE 5-2/1 and aluminum 2024-T3 was observed as differences of 10%.

Overall, aluminum 2024-T3 exhibited slightly better impact resistance than GLARE 5-2/1, as seen in Figure 8 and Figure 9. As shown in Table 4 of Guocai’s paper [6], the minimum cracking energy of aluminum 2024-T3 is a little higher than that of GLARE 5-2/1, and the particular energy to create a dent and crack damage in aluminum 2024-T3 is a little higher than GLARE 5-2/1. This is due to the effect of thickness and minimum cracking energy. However, in terms of impact energy dissipation, since GLARE 5-2/1 has the longer contact time than aluminum 2024-T3, it can absorb impact energy more and mitigate impact damage during a longer contact time. Additionally, GLARE can have an equivalent impact performance compared with aluminum 2024-T3 in terms of the relationship between the area density and impact energy.

### 4.2. Finite Element Simulation on Multiple Impact Behavior of GLARE 5-2/1

#### 4.2.1. Comparison of Experiments and FE Simulation

**Figure 11:**
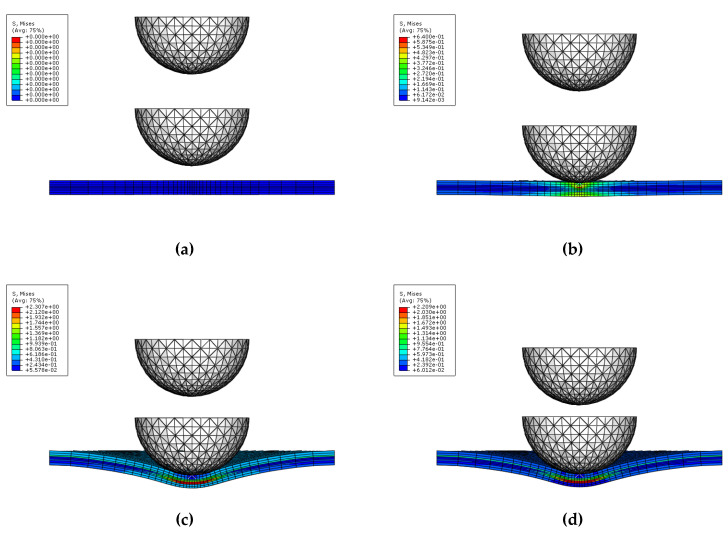
First impact applied on GLARE 5-2/1: (**a**) Time = 0 ms, (**b**) Time = 0.26 ms, (**c**) Time = 3.53 ms, (**d**) Time = 5.62 ms.

**Figure 12:**
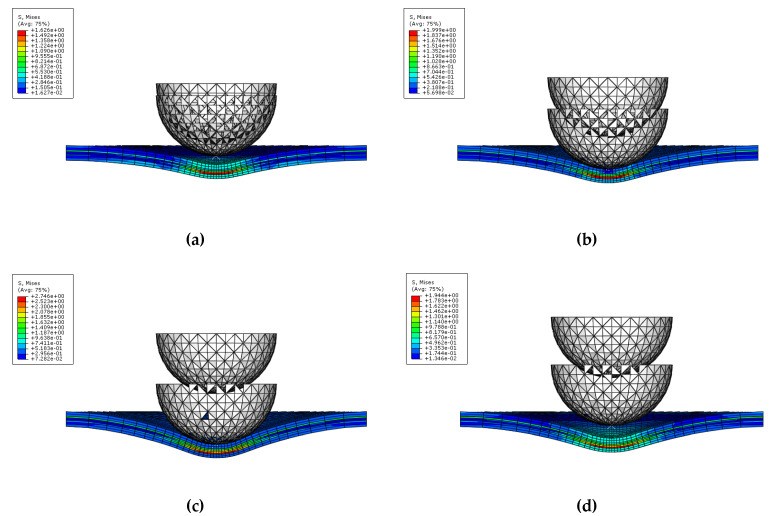
Second impact applied on GLARE 5-2/1: (**a**) Time = 0 ms, (**b**) Time = 0.26 ms, (**c**) Time = 2.22 ms, (**d**) Time = 5.35 ms.

**Figure 13:**
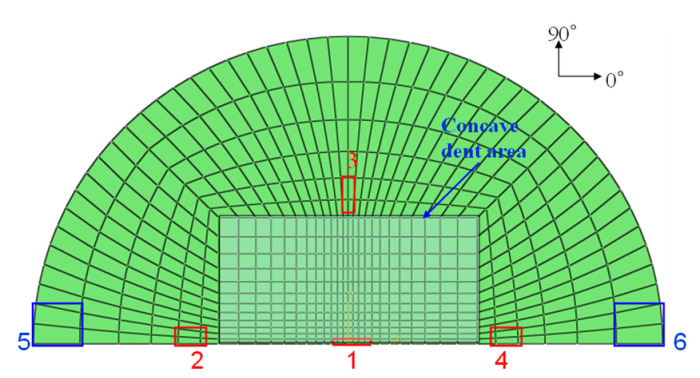
Detection of stress at different locations.

**Figure 14:**
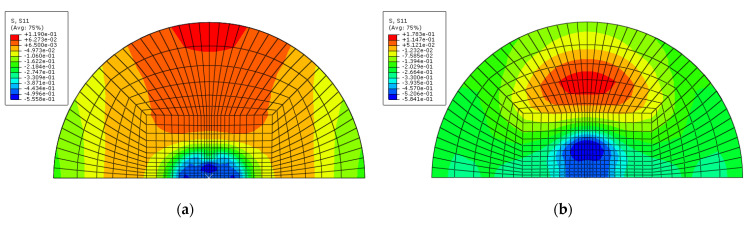

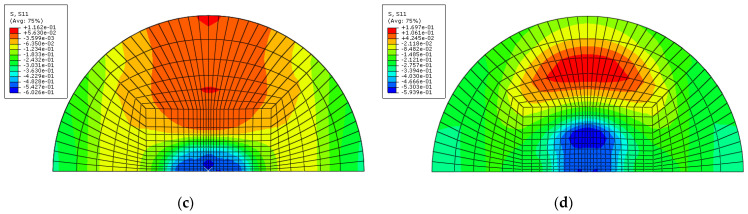
Detection of σ_11_ on impacted and non-impacted aluminum layer of GLARE 5-2/1: (**a**) first impact: impacted aluminum layer at 8 J, (**b**) first impact: non-impacted aluminum layer at 8 J, (**c**) second impact: impacted aluminum layer at 8 J and (**d**) second impact: non-impacted aluminum layer at 8 J.

**Figure 15:**
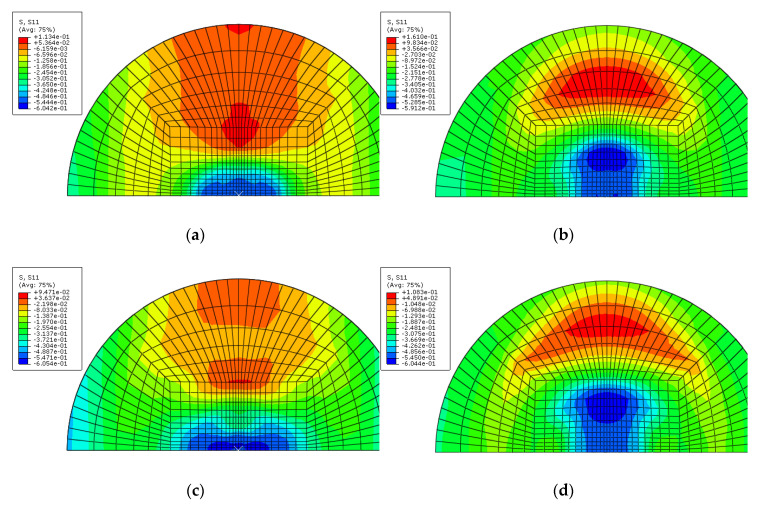
Detection of σ_11_ on impacted and non-impacted aluminum layer of GLARE 5-2/1: (**a**) first impact: impacted aluminum layer at 16 J, (**b**) first impact: non-impacted aluminum layer at 16 J, (**c**) second impact: impacted aluminum layer at 16 J and (**d**) second impact: non-impacted aluminum layer at 16 J.

Figure 11 and Figure 12 show the procedure for simulating multiple impact on GLARE 5-2/1. As seen in Figure 11 and Figure 12, the highest stress occurred in the composite layers compared to the aluminum layer after the first and second impacts. During the first and second impacts, the upper and lower sides of the composite layers were deformed by compressive load and tensile load, respectively. The highest stress that occurred was caused by the tensile load on the underside of the composite layers. Through low stress calculated on aluminum layer, it showed better impact resistance than the composite layers. As shown in Figure 13, the shaded area is assumed to be the concave dent area caused by multiple impact. The distance between locations 1 and location 3 is the same as the radius of the test impactor. Location 1 is assumed to be the first contact area, where the impactor impacted the GLARE laminate. As shown in Figure 11 and Figure 12, the GLARE laminate experienced a large deformation by multiple impact. To explore stress distribution by large deformation, the von-Misses stress and *σ*_11_ on impacted and non-impacted aluminum layer at six different points were investigated, respectively, as seen in Figure 14 and Figure 15. Table 5 and Table 6 summarize the von-Misses stress and *σ*_11_ detected on impacted and non-impacted surface aluminum layers of GLARE 5-2/1. At location 1 of the direct contact area for impact energies of 8 J and 16 J, the stress was higher than the tensile yield strength of aluminum. At clamped locations 5 and 6, the stress was higher than at locations 2–4 because of the fully fixed boundary condition. In the case of σ_11_ at an impact energy of 8 J, location 3 of the outer concave dent showed that tensile stress mainly dominated the impacted side. As shown in Figure 14 and Figure 15, the area of stress distribution was spread out widely on the impacted side but the stress on the non-impacted side was concentrated in a specific area. This caused the fracture to occur easily on the non-impacted side after applying the second impact.

**Figure 16:**
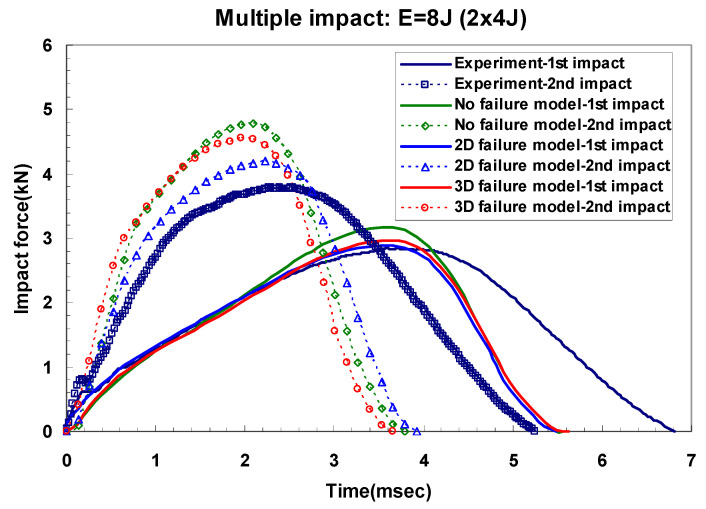
Multiple impact force-time curves. Comparison of experimental and simulation results for GLARE 5-2/1 under impact energy of 8 J.

**Figure 17:**
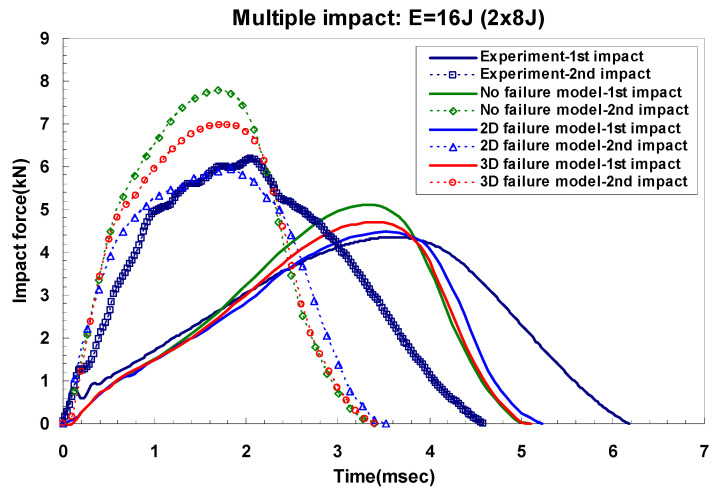
Multiple impact force-time curves. Comparison of experimental and simulation results for GLARE 5-2/1 under impact energy of 16 J.

Figure 16 and Figure 17 show a typical peak impact force and time history for GLARE 5-2/1. They also show comparisons of the non-failure model and the two- and three-dimensional failure models. In the case of the first impact, only plastic deformation in the aluminum layer was observed because low impact energies of 8 J and 16 J were applied. The first impact energy was almost absorbed by the aluminum layer rather than the composite layer [5,6]. As seen in Figure 16, the load-time history at 8 J after the 2nd impact showed the same trend as the first impact. Therefore, the two- and three-dimensional failure criteria in the composite layer did not critically degrade the stiffness of the GLARE laminate for multiple 8 J impacts. When multiple impacts were repeatedly made on the GLARE laminate, plastic deformation was only investigated in both experiments and FEM simulations if the applied impact energy was low. However, the dent damage was also created after the first impact at 16 J, but after the second impact, a different type of damage appeared. After applying the second impact, the composite layer experienced specific failure, as shown in the experimental data of Figure 17. This means that the stiffness of the composite layer started to degrade. As mentioned in the previous section on modeling in-plane degradation, the Hashin failure [17] damage model was used to set the appropriate individual laminate stiffness component to zero when maximum values were achieved in the longitudinal and transverse fiber or matrix tension criterion with a shearing strength form. As seen in Figure 17, the two- and three-dimensional Hashin failure criteria model to degrade the stiffness of the fiber-reinforced layer can improve the prediction of impact force and time history curve. It is clear that they were able to predict the impact response of the material. The prediction of the non-failure criteria model in terms of impact force and time history was overestimated when compared with experimental and failure criteria models, as shown in Figure 16 and Figure 17. The difference between the measured and predicted impact force is summarized in Table 7. Usually, the difference between the experiment and the simulation results is larger after the second impact than after the first impact. The critical reason for this discrepancy is believed to be the status of the boundary condition between the experiment and the finite element model. Additionally, the impact period is underestimated because of the perfect clamping assumption and one-directional constraint on the rigid impactor [31]. Overall, the finite element simulation results using a two- and three-dimensional failure model showed good agreement with experimental results.

**Figure 18:**
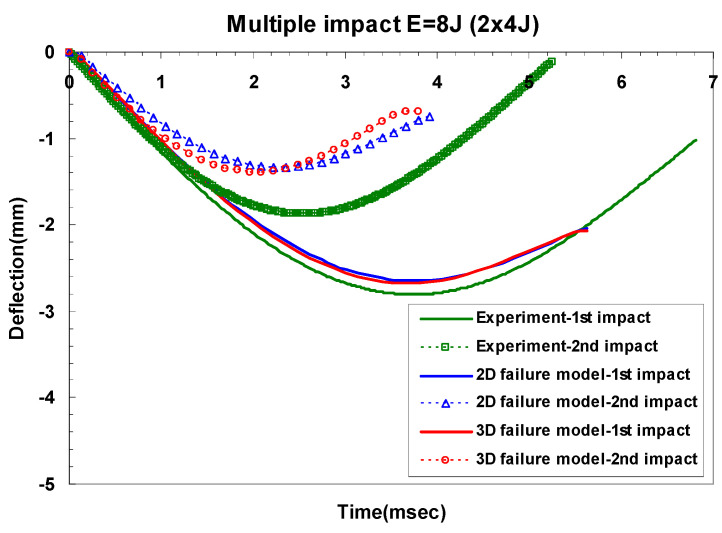
Difference between the predicted and measured central deflection for GLARE 5-2/1 under impact energy of 8 J.

**Figure 19:**
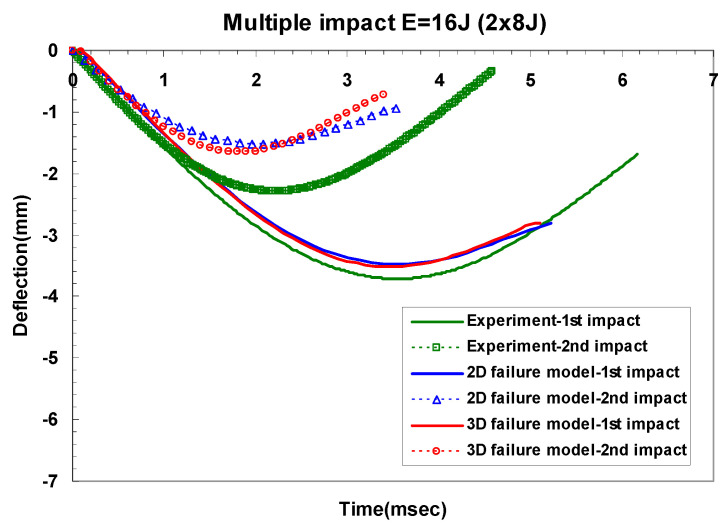
Difference between the predicted and measured central deflection for GLARE 5-2/1 under impact energy of 16 J.

Table 8 shows the difference of maximum dent depth for experiments and the two- and three-dimensional failure models in GLARE 5-2/1. As shown in Table 8, the two- and three-dimensional failure models showed almost similar dent depths. Additionally, the difference between experiment and simulation results exhibited a similar trend as the comparison of peak impact force. Figure 18 and Figure 19 show the changes of central deflection at different impact times. Because of the fully fixed boundary condition in the simulation, the finite element model of GLARE 5-2/1 was less deflected than the experimental one after the first and the second impacts. As shown in Figure 18 and Figure 19, there was no difference in the central deflection for the two- and three-dimensional failure model.

#### 4.2.2. Two- and Three-Dimensional Failure Criteria Comparison

**Figure 20:**
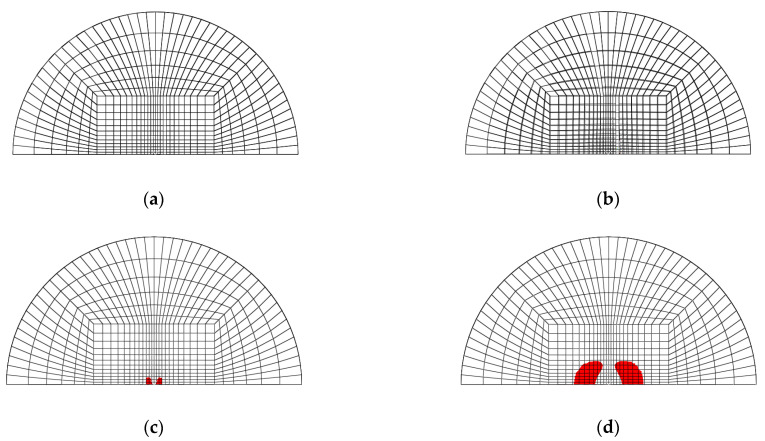
Fiber tension failure (FTF) in the composite layers of GLARE 5-2/1 with [0°/90°/90°/0°] after applying the first impact energy of 8 J; (**a**) 2D failure model (0.392 ms), (**b**) 2D failure model (3.66 ms), (**c**) 3D failure model (0.392 ms); (**d**) 3D failure model (3.66 ms).

**Figure 21:**
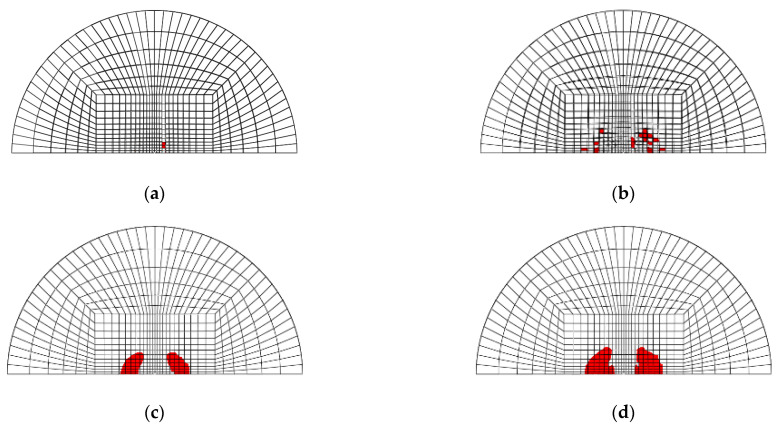
Fiber tension failure (FTF) in the composite layers of GLARE 5-2/1 with [0°/90°/90°/0°] after applying the second impact energy of 8 J; (**a**) 2D failure model (0.392 ms), (**b**) 2D failure model (2.22 ms), (**c**) 3D failure model (0.392 ms), (**d**) 3D failure model (1.96 ms).

**Figure 22:**
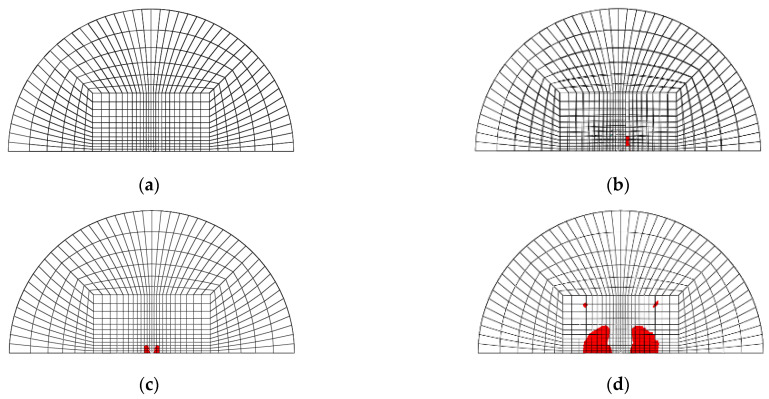
Fiber tension failure (FTF) in the composite layers of GLARE 5-2/1 with [0°/90°/90°/0°] after applying the first impact energy of 16 J; (**a**) 2D failure model (0.392 ms), (**b**) 2D failure model (3.53 ms), (**c**) 3D failure model (0.392 ms), (**d**) 3D failure model (3.4 ms).

**Figure 23:**
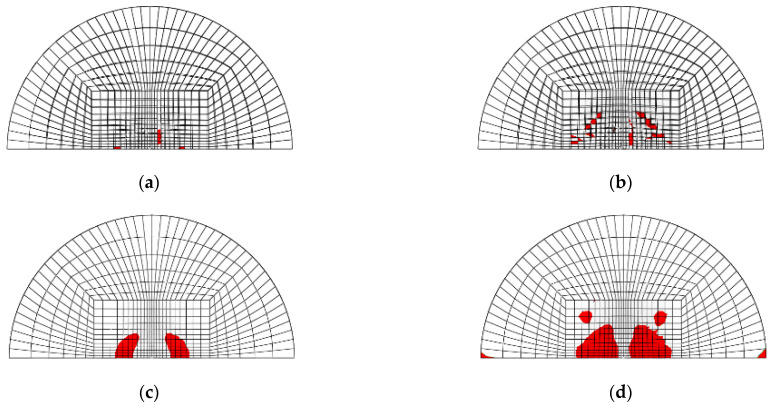
Fiber tension failure (FTF) in the composite layers of GLARE 5-2/1 with [0°/90°/90°/0°] after applying the second impact energy of 16 J; (**a**) 2D failure model (0.392 ms), (**b**) 2D failure model (1.83 ms), (**c**) 3D failure model (0.392 ms), (**d**) 3D failure model (1.96 ms).

Figure 20, Figure 21, Figure 22 and Figure 23 show the fiber damage progression in composite layers of GLARE 5-2/1 by multiple impact. Figure 20a,b, Figure 21a,b, Figure 22a,b and Figure 23a,b show the damage progression simulated by the two-dimensional failure model at multiple impact energies of 8 J and 16 J. Simulation results of the three-dimensional failure criterion model of 8 J and 16 J are shown in Figure 20c,d, Figure 21c,d, Figure 22c,d and Figure 23c,d. The colored damage was achieved by a state variable value greater than or equal to one, which corresponded to a point at which the failure criteria value was exceeded. The colored area displays the failure or damage of the fiber in the composite layer. As seen in each figure, the finite element model with three-dimensional failure criteria clearly had a larger number of integration points for damage initiation when compared with the two-dimensional failure criteria model.

## 5. Conclusions

In this paper, the multiple impact behaviors of aluminum 2024-T3 and GLARE 5-2/1 with diverse total impact energy were studied through experiments and finite element simulations. Three different multiple impact energies of 8 J (2 × 4 J), 16 J (2 × 8 J), and 26 J (2 × 13 J) were considered for aluminum 2024-T3 and GLARE 5-2/1 to create BVID and CVID. In the case of BVID at 8 J and 16 J, aluminum 2024-T3 and GLARE 5-2/1 showed plastic deformation dents on the outer aluminum layer. The CVID at 26 J for aluminum 2024-T3 was characterized by cracks from the plastic indentation in the fiber direction. In the case of GLARE 5-2/1, the combined damage of plastic deformation and failure at composite layers was investigated. This means that, due to the reduction of impact resistance of the aluminum layer, the impact energy was transferred to the composite layer and it caused the composite failure. After the first impact, the impact resistance of aluminum 2024-T3 and GLARE 5-2/1 was improved by increased stiffness attributed to the strain hardening of the aluminum layers. Additionally, through the similar slope value of stiffness of aluminum and GLAER, it was obvious that the aluminum layer had the critical contribution for impact behavior.

The finite element simulations were carried out on GLARE 5-2/1 for two different multiple impact energies of 8 J and 16 J. A three-dimensional failure model realized by VUMAT was compared with a two-dimensional failure model. Both failure models correlated well with experimental results. However, the two-dimensional failure model was better than the three-dimensional failure model for predicting peak impact force in multiple impact in the impact force-time history curve. The damage progression shape in the three-dimensional failure criteria model was larger than that of the two-dimensional criteria failure model.

## Figures and Tables

**Table 1:** Material properties used in the finite element model [4,5].

Material	Parameter Values, GPa					
	E_11_	E_22_	*G*_12_	G_23_	ν_12_	ν_23_
Al 2024-T3	72.2	72.2	-	-	0.33	0.33
S2 glass	55	9.5	5.5	3	0.33	0.33

**Table 2:** Damage and failure properties used in the finite element model [4,5].

Material	Parameter Values, MPa					
	σ_ys_	X_t_	X_c_	Y_t_	Y_c_	S_lt_
Al 2024-T3	320	-	-	-	-	-
S2 glass	-	2500	2000	50	150	75

**Table 3:** Degradation scheme for progressive composite damage [18].

Failure Mode	Degraded Elastic Material Properties				
	E_11_	E_22_	ν_12_	ν_23_	G_12_
Fiber tension (FT)	Χ	—	Χ	—	Χ
Fiber compression (FC)	Χ	—	Χ	—	Χ
Matrix tension/shear (MT)	—	Χ	Χ	Χ	Χ
Matrix compression (MC)	—	Χ	Χ	Χ	Χ

**Table 4:** Minimum cracking energy and perforation energy for GLARE laminates [6].

Materials	Thickness (mm)	Areal Density (kg/m^2^)	Minimum Cracking Energy (J)	Perforation Energy (J)
Aluminum 2024-T3	1.60	4.45	18.1	33.4
GLARE 5-2/1	1.56	3.74	16.3	34.5

**Table 5:** Von Mises stress on aluminum layer of GLARE 5-2/1.

Location	Stress (MPa)			
	8 J (Impacted)	8 J (Non-Impacted)	16 J (Impacted)	16 J (Non-Impacted)
1	515	531	531	532
2	140	254	328	226
3	137	252	320	242
4	141	248	329	224
5	343	268	307	307
6	344	266	303	303

**Table 6:** *σ*_11_ on aluminum layer of GLARE 5-2/1.

Location	Stress (MPa)			
	8 J (Impacted)	8 J (Non-Impacted)	16 J (Impacted)	16 J (Non-Impacted)
1	−528	−522	−548	−518
2	−111	−228	−303	−227
3	56	10	−4	−146
4	−112	−223	−309	−223
5	−290	−288	−398	−305
6	−292	−286	−397	−299

**Table 7:** Difference between predicted and measured peak impact force for GLARE 5-2/1.

Impact Energy	Differences of Peak Impact Force (%)			
	2D Failure Model		3D Failure Model	
	First Impact	Second Impact	First Impact	Second Impact
8 J	1.41	11.1	4.58	20.37
16 J	2.75	1.00	8.02	16.14

**Table 8:** Difference between the predicted and measured maximum dent depth for GLARE 5-2/1.

Impact Energy	Differences of Maximum Dent Depth (%)			
	2D Failure Model		3D Failure Model	
	First Impact	Second Impact	First Impact	Second Impact
8 J	6.69	30.0	5.63	26.8
16 J	7.47	34.8	6.40	29.2

## Data Availability

Data is contained within the article.

## Nomenclature

$\sigma_{ij}$	Material stress state
$\varepsilon_{ij}$	Material strain state
$\varepsilon_{ij+}{}^{init}$	Tensile strain component at failure
$\varepsilon_{ij-}{}^{init}$	Compressive strain component at failure
$f^{ft}$	Hashin fiber tension failure initiation
$f^{fc}$	Hashin fiber compression failure initiation
$f^{mt}$	Hashin matrix tension failure initiation
$f^{mc}$	Hashin matrix compression failure initiation
$E_{11}$	Composite modulus along the fiber axis
$E_{22}$	Composite modulus transverse to the fiber axis
$\upsilon_{12}$	Poisson’s ratio for the fiber-matrix direction
$\upsilon_{23}$	Poisson’s ratio for the matrix-matrix direction
$G_{12}$	Shear modulus fiber-matrix direction
t	Time value at current finite element analysis increment
Δt	Time step between successive increments
$\dot{w^{i}}$	Damage propagation rate for each failure mode (i)
$w^{i}$	Damage state variable for each failure mode (i)
$\Omega_{0}$	Damage nucleation rate constant
$\Omega_{1}$	Damage growth rate constant
$S_{ij}$	Material compliance matrix
d	Material degradation factor
$C_{ij}$	Material stiffness matrix
